# Diethyl [(2-bromo­anilino)(1,3-diphenyl-1*H*-pyrazol-4-yl)meth­yl]phospho­nate

**DOI:** 10.1107/S1600536812017849

**Published:** 2012-04-28

**Authors:** G. Suresh, V. Sabari, A. Nandakumar, P. T. Perumal, S. Aravindhan

**Affiliations:** a Department of Physics, Presidency college (Autonomous), Chennai 600 005, India; b Organic Chemistry Laboratory, CLRI, Chennai, Tamil Nadu, India

## Abstract

In the title compound, C_26_H_27_BrN_3_O_3_P, the central pyrazole ring forms a dihedral angle of 71.7 (2)° with the bromo­phenyl ring. In the crystal, mol­ecules are linked by pairs of N—H⋯O hydrogen bonds, forming inversion dimers with *R*_2_^2^(10) ring motifs. Four C atoms of the 3-phenyl ring are disordered over two sets of sites [site occupancies = 0.745 (6) and 0.225 (6)].

## Related literature

For information on pyrazole derivatives, see: Sullivan *et al.* (2006 ▶); Patel *et al.* (2010 ▶). For a related structure, see: Saeed *et al.* (2009 ▶).
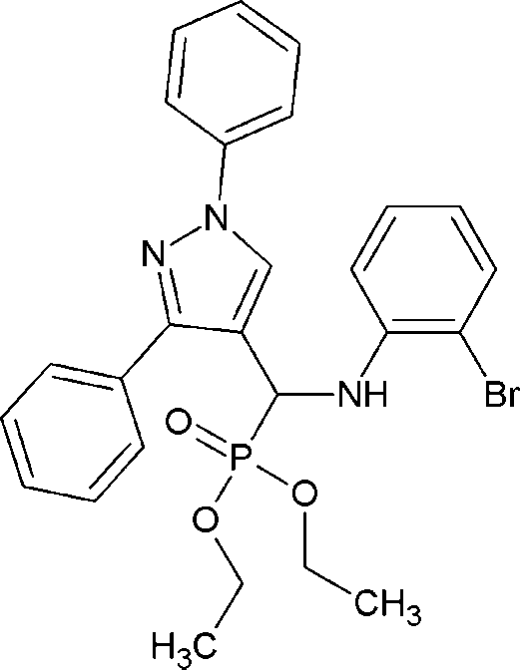


## Experimental

### 

#### Crystal data

C_26_H_27_BrN_3_O_3_P
*M* *_r_* = 540.39
Monoclinic, 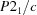

*a* = 11.2553 (6) Å
*b* = 23.9104 (15) Å
*c* = 9.4741 (5) Å
β = 91.229 (3)°
*V* = 2549.1 (2) Å^3^
*Z* = 4
Mo *K*α radiation
μ = 1.71 mm^−1^
*T* = 293 K
0.25 × 0.22 × 0.19 mm

#### Data collection

Bruker APEXII CCD area-detector diffractometer
Absorption correction: multi-scan (*SADABS*; Bruker, 2004 ▶) *T*_min_ = 0.953, *T*_max_ = 0.964
35555 measured reflections
4502 independent reflections
3093 reflections with *I* > 2σ(*I*)
*R*_int_ = 0.065

#### Refinement

*R*[*F* ^2^ > 2σ(*F* ^2^)] = 0.047
*wR*(*F* ^2^) = 0.129
*S* = 1.09
4502 reflections
346 parameters
H-atom parameters constrained
Δρ_max_ = 0.63 e Å^−3^
Δρ_min_ = −0.34 e Å^−3^

### 

Data collection: *APEX2* (Bruker, 2004 ▶); cell refinement: *SAINT* (Bruker, 2004 ▶); data reduction: *SAINT*; program(s) used to solve structure: *SHELXS97* (Sheldrick, 2008 ▶); program(s) used to refine structure: *SHELXL97* (Sheldrick, 2008 ▶); molecular graphics: *ORTEP-3 for Windows* (Farrugia, 1997 ▶); software used to prepare material for publication: *PLATON* (Spek, 2009 ▶).

## Supplementary Material

Crystal structure: contains datablock(s) I, global. DOI: 10.1107/S1600536812017849/bt5876sup1.cif

Structure factors: contains datablock(s) I. DOI: 10.1107/S1600536812017849/bt5876Isup2.hkl

Supplementary material file. DOI: 10.1107/S1600536812017849/bt5876Isup3.cml

Additional supplementary materials:  crystallographic information; 3D view; checkCIF report

## Figures and Tables

**Table 1:** Hydrogen-bond geometry (Å, °)

*D*—H⋯*A*	*D*—H	H⋯*A*	*D*⋯*A*	*D*—H⋯*A*
N1—H1⋯O1^i^	0.86	2.48	3.305 (4)	162

Symmetry code: (i) 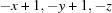.

**Fig. 1.**
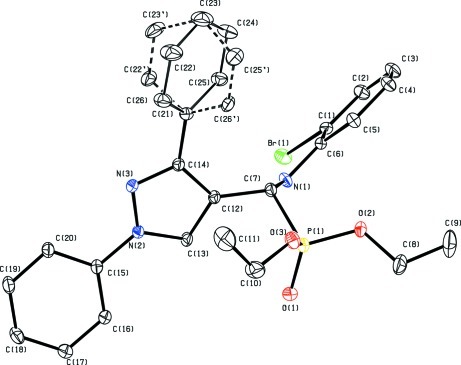
The molecular structure of the title compound, showing 10% probability displacement ellipsoids for non-H atoms.

**Fig. 2.**
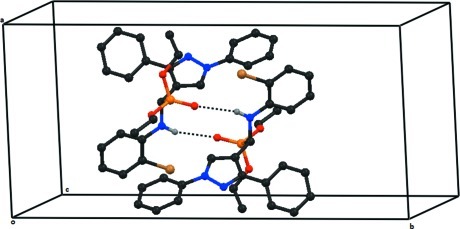
Partial crystal packing diagram of the title compound. H atoms not involved in hydrogen bonding (dashed lines) have been omitted for clarity.

## supplementary crystallographic information

### Comment

Pyrazoles exhibit a variety of pharmacological properties for e.g antibacterial and anti-inflammatory activities (Sullivan *et al.*, 2006; Patel *et al.*, 2010). In view of their importance, the crystal structure determination of the title compound was carried out and the results are presented here.

X-Ray analysis confirms the molecular structure and atom connectivity as illustrated in Fig. 1. The bond lengths N2—C13 and N3—C14 are normal and comparable to the corresponding values observed in the related structure of 3-(3-Chloroanilino)-1-(3,5-dimethyl-1*H*-pyrazol-1-yl)propan-1-one (Saeed *et al.*, 2009). The central pyrazole ring and the bromophenyl ring are almost perpendicular with the dihedral angle of 71.7 (2) °, whereas the two phenyl rings are twisted from the pyrazole ring as can be seen from the dihedral angle of 15.0 (1)° and 39.3 (3)°, respectively. The pyrazole ring system is essentially planar, with maximum deviation of 0.006 (4) for atom C14. The sum of bond angles around N2[359.7 (3) °] of the pyrazole ring is in accordance with *sp*^3^ hybridization. The atoms Br1 and N1 are deviated by -0.039 (1) Å and 0.019 (3) Å from the leastsquares plane of the benzene(C1—C6) ring. The four carbon atoms in the phenyl ring are disordered over two sets of sites [site occupancies = 0.745 (6) and 0.225 (6)].

The phosphinite group assumes an extended conformation as can be seen from torsion angles P1—O2—C8—C9 of -179.0 (5)°. A pair of N1—H1···O1 hydrogen bonds at *x*,*y*,*z* and -*x*,1 - *y*,2 - *z* form a cyclic centrosymmetric dimer [*R*^2^_2_(10)]. These hdrogen bonds are crosslinked to Br1 to form S(5) ring (C1—C6—N1—H1—Br1) motif. The crystal packing is stabilized N—H···O hydrogen bonds.

### Experimental

A mixture of 1,3-diphenyl-1*H*-pyrazole-4-carbaldehyde (1 mmol), 2-bromoaniline (1 mmol), diethyl phosphite (1.5 mmol), and pottasium hydrogen sulfate (20 mol-%) under neat condition was stirred at room temperature. After completion of the reaction as indicated by TLC, it was poured into water and extracted with ethyl acetate. The organic layer was dried over sodium sulfate and concentrated under vacuum. The crude product was chromatographed.

### Refinement

The four carbon atoms in one of the phenyl ring are disordered over two positions (C22/C22', C23/C23',C25/C25'and C26/C26') with refined occupancies of 0.745 (6) and 0.225 (6). The corresponding bond distances involving the disordered atoms were restrained to be equal. All H atoms were fixed geometrically and allowed to ride on their parent C atoms, with C—H distances fixed in the range 0.93–0.97 Å with *U*_iso_(H) = 1.5*U*_eq_(C) for methyl H 1.2*U*_eq_(C) for other H atoms.

### Crystal data

**Table:**

C_26_H_27_BrN_3_O_3_P	*F*(000) = 1112
*M**_r_* = 540.39	*D*_x_ = 1.408 Mg m^−^^3^
Monoclinic, *P*2_1_/*c*	Mo *K*α radiation, λ = 0.71073 Å
Hall symbol: -P 2ybc	Cell parameters from 4502 reflections
*a* = 11.2553 (6) Å	θ = 1.8–25.0°
*b* = 23.9104 (15) Å	µ = 1.71 mm^−^^1^
*c* = 9.4741 (5) Å	*T* = 293 K
β = 91.229 (3)°	Block, colourless
*V* = 2549.1 (2) Å^3^	0.25 × 0.22 × 0.19 mm
*Z* = 4	

### Data collection

**Table:**

Bruker APEXII CCD area-detector diffractometer	4502 independent reflections
Radiation source: fine-focus sealed tube	3093 reflections with *I* > 2σ(*I*)
Graphite monochromator	*R*_int_ = 0.065
ω and φ scans	θ_max_ = 25.0°, θ_min_ = 1.8°
Absorption correction: multi-scan (*SADABS*; Bruker, 2004)	*h* = −13→13
*T*_min_ = 0.953, *T*_max_ = 0.964	*k* = −28→27
35555 measured reflections	*l* = −11→11

### Refinement

**Table:**

Refinement on *F*^2^	Primary atom site location: structure-invariant direct methods
Least-squares matrix: full	Secondary atom site location: difference Fourier map
*R*[*F*^2^ > 2σ(*F*^2^)] = 0.047	Hydrogen site location: inferred from neighbouring sites
*wR*(*F*^2^) = 0.129	H-atom parameters constrained
*S* = 1.09	*w* = 1/[σ^2^(*F*_o_^2^) + (0.0498*P*)^2^ + 2.1289*P*] where *P* = (*F*_o_^2^ + 2*F*_c_^2^)/3
4502 reflections	(Δ/σ)_max_ = 0.001
346 parameters	Δρ_max_ = 0.63 e Å^−^^3^
0 restraints	Δρ_min_ = −0.34 e Å^−^^3^

### Special details

**Table:**

Geometry. All e.s.d.'s (except the e.s.d. in the dihedral angle between two l.s. planes) are estimated using the full covariance matrix. The cell e.s.d.'s are taken into account individually in the estimation of e.s.d.'s in distances, angles and torsion angles; correlations between e.s.d.'s in cell parameters are only used when they are defined by crystal symmetry. An approximate (isotropic) treatment of cell e.s.d.'s is used for estimating e.s.d.'s involving l.s. planes.
Refinement. Refinement of *F*^2^ against ALL reflections. The weighted *R*-factor *wR* and goodness of fit *S* are based on *F*^2^, conventional *R*-factors *R* are based on *F*, with *F* set to zero for negative *F*^2^. The threshold expression of *F*^2^ > σ(*F*^2^) is used only for calculating *R*-factors(gt) *etc*. and is not relevant to the choice of reflections for refinement. *R*-factors based on *F*^2^ are statistically about twice as large as those based on *F*, and *R*- factors based on ALL data will be even larger.

### Fractional atomic coordinates and isotropic or equivalent isotropic displacement parameters (Å^2^)

**Table:**

	*x*	*y*	*z*	*U*_iso_*/*U*_eq_	Occ. (<1)
C1	0.7034 (4)	0.66776 (16)	−0.0245 (4)	0.0574 (10)	
C2	0.7722 (4)	0.7133 (2)	0.0147 (5)	0.0779 (13)	
H2	0.8481	0.7177	−0.0207	0.093*	
C3	0.7276 (5)	0.7521 (2)	0.1063 (5)	0.0865 (15)	
H3	0.7733	0.7829	0.1329	0.104*	
C4	0.6169 (5)	0.74563 (19)	0.1580 (5)	0.0781 (13)	
H4	0.5867	0.7723	0.2191	0.094*	
C5	0.5490 (4)	0.69993 (18)	0.1209 (4)	0.0673 (11)	
H5	0.4742	0.6958	0.1593	0.081*	
C6	0.5898 (3)	0.65948 (15)	0.0268 (4)	0.0511 (9)	
C7	0.3974 (3)	0.60774 (16)	0.0142 (4)	0.0505 (9)	
H7	0.3663	0.6453	0.0315	0.061*	
C8	0.4361 (6)	0.5769 (3)	0.4318 (5)	0.120 (2)	
H8A	0.3557	0.5708	0.4634	0.143*	
H8B	0.4762	0.5410	0.4315	0.143*	
C9	0.4960 (6)	0.6131 (3)	0.5283 (6)	0.143 (3)	
H9A	0.5745	0.6206	0.4955	0.214*	
H9B	0.5012	0.5956	0.6195	0.214*	
H9C	0.4527	0.6476	0.5353	0.214*	
C10	0.1356 (5)	0.5406 (2)	0.1624 (7)	0.1005 (18)	
H10A	0.1640	0.5114	0.1007	0.121*	
H10B	0.1072	0.5230	0.2476	0.121*	
C11	0.0402 (5)	0.5692 (3)	0.0941 (8)	0.136 (3)	
H11A	0.0227	0.6027	0.1455	0.204*	
H11B	−0.0287	0.5455	0.0909	0.204*	
H11C	0.0620	0.5788	−0.0002	0.204*	
C12	0.3294 (3)	0.58391 (16)	−0.1095 (4)	0.0468 (8)	
C13	0.3284 (3)	0.52928 (17)	−0.1523 (4)	0.0530 (9)	
H13	0.3733	0.5004	−0.1127	0.064*	
C14	0.2477 (3)	0.61084 (16)	−0.2020 (4)	0.0482 (9)	
C15	0.2134 (3)	0.47520 (16)	−0.3328 (4)	0.0490 (9)	
C16	0.2418 (4)	0.42335 (18)	−0.2799 (5)	0.0724 (13)	
H16	0.2901	0.4204	−0.1994	0.087*	
C17	0.1996 (5)	0.37591 (19)	−0.3447 (5)	0.0813 (14)	
H17	0.2201	0.3410	−0.3082	0.098*	
C18	0.1276 (4)	0.3795 (2)	−0.4627 (5)	0.0758 (13)	
H18	0.0960	0.3474	−0.5042	0.091*	
C19	0.1031 (4)	0.4311 (2)	−0.5184 (5)	0.0775 (14)	
H19	0.0564	0.4337	−0.6004	0.093*	
C20	0.1461 (4)	0.47938 (19)	−0.4557 (4)	0.0695 (12)	
H20	0.1300	0.5141	−0.4958	0.083*	
C24	0.1410 (7)	0.7813 (3)	−0.2076 (8)	0.115 (2)	
H24	0.1173	0.8185	−0.2103	0.138*	
C21	0.2108 (3)	0.67015 (16)	−0.2048 (4)	0.0568 (10)	
C22	0.2954 (5)	0.7121 (2)	−0.1798 (6)	0.0693 (17)	0.745 (6)
H22	0.3746	0.7027	−0.1627	0.083*	0.745 (6)
C23	0.2609 (7)	0.7677 (3)	−0.1808 (8)	0.092 (2)	0.745 (6)
H23	0.3165	0.7958	−0.1639	0.110*	0.745 (6)
C25	0.0615 (8)	0.7409 (4)	−0.2291 (12)	0.114 (3)	0.745 (6)
H25	−0.0179	0.7502	−0.2445	0.137*	0.745 (6)
C26	0.0949 (6)	0.6850 (3)	−0.2290 (8)	0.082 (2)	0.745 (6)
H26	0.0380	0.6574	−0.2455	0.098*	0.745 (6)
C22'	0.178 (2)	0.6952 (8)	−0.3278 (19)	0.083 (7)	0.255 (6)
H22'	0.1787	0.6743	−0.4106	0.100*	0.255 (6)
C23'	0.143 (3)	0.7510 (10)	−0.335 (2)	0.104 (9)	0.255 (6)
H23'	0.1223	0.7676	−0.4205	0.125*	0.255 (6)
C25'	0.144 (2)	0.7541 (9)	−0.083 (2)	0.093 (7)	0.255 (6)
H25'	0.1212	0.7723	−0.0014	0.111*	0.255 (6)
C26'	0.1800 (15)	0.6992 (7)	−0.0784 (16)	0.067 (5)	0.255 (6)
H26'	0.1846	0.6808	0.0080	0.080*	0.255 (6)
N1	0.5235 (3)	0.61313 (13)	−0.0113 (3)	0.0555 (8)	
H1	0.5589	0.5860	−0.0525	0.067*	
N2	0.2510 (2)	0.52470 (13)	−0.2618 (3)	0.0488 (7)	
N3	0.1990 (3)	0.57471 (13)	−0.2936 (3)	0.0514 (7)	
O1	0.3995 (3)	0.50661 (12)	0.1577 (3)	0.0676 (8)	
O2	0.4314 (3)	0.59874 (12)	0.2902 (3)	0.0672 (8)	
O3	0.2326 (2)	0.57768 (13)	0.1987 (3)	0.0731 (8)	
P1	0.36718 (9)	0.56534 (5)	0.16934 (10)	0.0536 (3)	
Br1	0.76574 (4)	0.61592 (2)	−0.15402 (5)	0.0757 (2)	

### Atomic displacement parameters (Å^2^)

**Table:**

	*U*^11^	*U*^22^	*U*^33^	*U*^12^	*U*^13^	*U*^23^
C1	0.068 (2)	0.048 (2)	0.056 (2)	−0.013 (2)	−0.0117 (19)	0.0093 (18)
C2	0.083 (3)	0.068 (3)	0.082 (3)	−0.033 (3)	−0.004 (2)	0.005 (3)
C3	0.116 (4)	0.062 (3)	0.081 (3)	−0.038 (3)	−0.007 (3)	−0.004 (3)
C4	0.107 (4)	0.051 (3)	0.075 (3)	−0.011 (3)	−0.005 (3)	−0.014 (2)
C5	0.078 (3)	0.057 (3)	0.066 (3)	−0.008 (2)	−0.007 (2)	−0.010 (2)
C6	0.063 (2)	0.041 (2)	0.049 (2)	−0.0047 (18)	−0.0167 (17)	0.0025 (17)
C7	0.048 (2)	0.051 (2)	0.051 (2)	−0.0002 (17)	−0.0102 (16)	−0.0067 (17)
C8	0.167 (6)	0.137 (6)	0.053 (3)	−0.051 (5)	−0.024 (3)	0.012 (3)
C9	0.139 (6)	0.229 (9)	0.060 (3)	−0.058 (5)	−0.014 (3)	−0.022 (4)
C10	0.078 (3)	0.093 (4)	0.130 (5)	−0.024 (3)	−0.012 (3)	0.019 (3)
C11	0.062 (3)	0.153 (7)	0.193 (7)	−0.007 (4)	−0.022 (4)	−0.021 (5)
C12	0.0424 (19)	0.051 (2)	0.0463 (19)	−0.0025 (17)	−0.0058 (15)	−0.0005 (17)
C13	0.051 (2)	0.056 (3)	0.051 (2)	0.0059 (18)	−0.0178 (17)	−0.0033 (18)
C14	0.0476 (19)	0.052 (2)	0.0448 (19)	−0.0049 (17)	−0.0047 (15)	0.0027 (17)
C15	0.047 (2)	0.056 (2)	0.044 (2)	0.0036 (17)	−0.0028 (16)	−0.0077 (17)
C16	0.097 (3)	0.056 (3)	0.062 (3)	0.000 (2)	−0.031 (2)	0.002 (2)
C17	0.109 (4)	0.052 (3)	0.082 (3)	0.001 (2)	−0.026 (3)	−0.002 (2)
C18	0.082 (3)	0.063 (3)	0.082 (3)	0.000 (2)	−0.008 (2)	−0.027 (2)
C19	0.091 (3)	0.071 (3)	0.069 (3)	0.014 (3)	−0.031 (2)	−0.025 (2)
C20	0.086 (3)	0.058 (3)	0.063 (3)	0.014 (2)	−0.029 (2)	−0.012 (2)
C24	0.136 (6)	0.055 (4)	0.152 (7)	0.018 (4)	−0.006 (5)	0.009 (4)
C21	0.062 (2)	0.047 (2)	0.060 (2)	−0.0024 (19)	−0.0069 (19)	0.0054 (19)
C22	0.069 (4)	0.058 (4)	0.081 (4)	−0.009 (3)	0.003 (3)	0.009 (3)
C23	0.102 (6)	0.050 (4)	0.123 (6)	−0.015 (4)	0.008 (5)	0.003 (4)
C25	0.091 (5)	0.071 (6)	0.178 (10)	0.019 (5)	−0.029 (6)	0.019 (5)
C26	0.069 (4)	0.056 (4)	0.118 (6)	0.000 (3)	−0.019 (4)	0.008 (4)
C22'	0.127 (18)	0.060 (12)	0.062 (11)	0.047 (12)	−0.016 (11)	−0.005 (9)
C23'	0.16 (2)	0.079 (15)	0.069 (13)	0.057 (16)	0.003 (14)	0.018 (12)
C25'	0.130 (19)	0.080 (14)	0.068 (12)	0.049 (13)	0.008 (12)	−0.011 (11)
C26'	0.084 (12)	0.072 (12)	0.045 (9)	0.018 (9)	−0.012 (8)	0.004 (8)
N1	0.0503 (17)	0.0497 (19)	0.066 (2)	−0.0076 (15)	−0.0058 (14)	−0.0149 (16)
N2	0.0516 (17)	0.0470 (19)	0.0474 (17)	0.0032 (14)	−0.0117 (13)	−0.0028 (14)
N3	0.0570 (18)	0.0499 (19)	0.0467 (16)	0.0017 (15)	−0.0124 (14)	0.0023 (15)
O1	0.0795 (19)	0.0542 (18)	0.0686 (17)	0.0002 (14)	−0.0087 (14)	0.0044 (14)
O2	0.0795 (19)	0.0733 (19)	0.0479 (15)	−0.0120 (15)	−0.0163 (13)	−0.0021 (13)
O3	0.0599 (17)	0.080 (2)	0.0793 (19)	−0.0114 (15)	−0.0013 (14)	−0.0111 (16)
P1	0.0526 (5)	0.0572 (7)	0.0506 (6)	−0.0040 (5)	−0.0092 (4)	−0.0032 (5)
Br1	0.0700 (3)	0.0664 (3)	0.0911 (4)	−0.0081 (2)	0.0090 (2)	−0.0043 (2)

### Geometric parameters (Å, º)

**Table:**

C1—C2	1.383 (6)	C15—C20	1.379 (5)
C1—C6	1.392 (5)	C15—N2	1.422 (5)
C1—Br1	1.890 (4)	C16—C17	1.370 (6)
C2—C3	1.372 (7)	C16—H16	0.9300
C2—H2	0.9300	C17—C18	1.369 (6)
C3—C4	1.358 (7)	C17—H17	0.9300
C3—H3	0.9300	C18—C19	1.368 (6)
C4—C5	1.374 (6)	C18—H18	0.9300
C4—H4	0.9300	C19—C20	1.382 (6)
C5—C6	1.400 (6)	C19—H19	0.9300
C5—H5	0.9300	C20—H20	0.9300
C6—N1	1.380 (4)	C24—C25	1.330 (10)
C7—N1	1.451 (5)	C24—C25'	1.34 (2)
C7—C12	1.498 (5)	C24—C23	1.406 (9)
C7—P1	1.824 (4)	C24—C23'	1.41 (2)
C7—H7	0.9800	C24—H24	0.9300
C8—C9	1.419 (8)	C21—C22'	1.355 (17)
C8—O2	1.439 (5)	C21—C26	1.366 (7)
C8—H8A	0.9700	C21—C22	1.400 (7)
C8—H8B	0.9700	C21—C26'	1.434 (16)
C9—H9A	0.9600	C22—C23	1.384 (9)
C9—H9B	0.9600	C22—H22	0.9300
C9—H9C	0.9600	C23—H23	0.9300
C10—C11	1.417 (8)	C25—C26	1.388 (10)
C10—O3	1.442 (5)	C25—H25	0.9300
C10—H10A	0.9700	C26—H26	0.9300
C10—H10B	0.9700	C22'—C23'	1.39 (3)
C11—H11A	0.9600	C22'—H22'	0.9300
C11—H11B	0.9600	C23'—H23'	0.9300
C11—H11C	0.9600	C25'—C26'	1.38 (2)
C12—C13	1.368 (5)	C25'—H25'	0.9300
C12—C14	1.412 (5)	C26'—H26'	0.9300
C13—N2	1.344 (4)	N1—H1	0.8600
C13—H13	0.9300	N2—N3	1.362 (4)
C14—N3	1.334 (4)	O1—P1	1.455 (3)
C14—C21	1.478 (5)	O2—P1	1.561 (3)
C15—C16	1.373 (6)	O3—P1	1.574 (3)
			
C2—C1—C6	122.1 (4)	C19—C18—H18	120.6
C2—C1—Br1	118.5 (4)	C17—C18—H18	120.6
C6—C1—Br1	119.3 (3)	C18—C19—C20	121.5 (4)
C3—C2—C1	119.5 (5)	C18—C19—H19	119.3
C3—C2—H2	120.3	C20—C19—H19	119.3
C1—C2—H2	120.3	C15—C20—C19	118.9 (4)
C4—C3—C2	120.1 (4)	C15—C20—H20	120.5
C4—C3—H3	119.9	C19—C20—H20	120.5
C2—C3—H3	119.9	C25—C24—C25'	77.6 (12)
C3—C4—C5	120.5 (5)	C25—C24—C23	120.0 (6)
C3—C4—H4	119.7	C25'—C24—C23	74.1 (11)
C5—C4—H4	119.7	C25—C24—C23'	61.2 (12)
C4—C5—C6	121.6 (4)	C25'—C24—C23'	120.1 (13)
C4—C5—H5	119.2	C23—C24—C23'	90.0 (12)
C6—C5—H5	119.2	C25—C24—H24	120.0
N1—C6—C1	121.2 (4)	C25'—C24—H24	119.2
N1—C6—C5	122.6 (4)	C23—C24—H24	120.0
C1—C6—C5	116.1 (4)	C23'—C24—H24	118.7
N1—C7—C12	112.8 (3)	C22'—C21—C26	60.0 (10)
N1—C7—P1	112.6 (2)	C22'—C21—C22	90.0 (11)
C12—C7—P1	108.5 (2)	C26—C21—C22	119.1 (5)
N1—C7—H7	107.5	C22'—C21—C26'	116.0 (10)
C12—C7—H7	107.5	C26—C21—C26'	76.5 (7)
P1—C7—H7	107.5	C22—C21—C26'	71.8 (8)
C9—C8—O2	112.7 (5)	C22'—C21—C14	120.7 (8)
C9—C8—H8A	109.0	C26—C21—C14	121.2 (4)
O2—C8—H8A	109.0	C22—C21—C14	119.7 (4)
C9—C8—H8B	109.0	C26'—C21—C14	121.6 (7)
O2—C8—H8B	109.0	C23—C22—C21	119.8 (6)
H8A—C8—H8B	107.8	C23—C22—H22	120.1
C8—C9—H9A	109.5	C21—C22—H22	120.1
C8—C9—H9B	109.5	C22—C23—C24	119.4 (6)
H9A—C9—H9B	109.5	C22—C23—H23	120.3
C8—C9—H9C	109.5	C24—C23—H23	120.3
H9A—C9—H9C	109.5	C24—C25—C26	121.2 (7)
H9B—C9—H9C	109.5	C24—C25—H25	119.4
C11—C10—O3	112.0 (5)	C26—C25—H25	119.4
C11—C10—H10A	109.2	C21—C26—C25	120.5 (6)
O3—C10—H10A	109.2	C21—C26—H26	119.8
C11—C10—H10B	109.2	C25—C26—H26	119.8
O3—C10—H10B	109.2	C21—C22'—C23'	122.4 (16)
H10A—C10—H10B	107.9	C21—C22'—H22'	118.8
C10—C11—H11A	109.5	C23'—C22'—H22'	118.8
C10—C11—H11B	109.5	C22'—C23'—C24	117.6 (16)
H11A—C11—H11B	109.5	C22'—C23'—H23'	121.2
C10—C11—H11C	109.5	C24—C23'—H23'	121.2
H11A—C11—H11C	109.5	C24—C25'—C26'	119.5 (15)
H11B—C11—H11C	109.5	C24—C25'—H25'	120.2
C13—C12—C14	104.5 (3)	C26'—C25'—H25'	120.2
C13—C12—C7	126.6 (3)	C25'—C26'—C21	120.7 (14)
C14—C12—C7	128.8 (3)	C25'—C26'—H26'	119.6
N2—C13—C12	107.9 (3)	C21—C26'—H26'	119.6
N2—C13—H13	126.0	C6—N1—C7	123.6 (3)
C12—C13—H13	126.0	C6—N1—H1	118.2
N3—C14—C12	111.2 (3)	C7—N1—H1	118.2
N3—C14—C21	119.9 (3)	C13—N2—N3	111.6 (3)
C12—C14—C21	128.9 (3)	C13—N2—C15	127.9 (3)
C16—C15—C20	119.6 (4)	N3—N2—C15	120.2 (3)
C16—C15—N2	120.9 (3)	C14—N3—N2	104.8 (3)
C20—C15—N2	119.5 (3)	C8—O2—P1	120.3 (3)
C17—C16—C15	120.6 (4)	C10—O3—P1	124.7 (3)
C17—C16—H16	119.7	O1—P1—O2	115.90 (17)
C15—C16—H16	119.7	O1—P1—O3	115.93 (18)
C18—C17—C16	120.5 (4)	O2—P1—O3	101.93 (16)
C18—C17—H17	119.7	O1—P1—C7	115.09 (17)
C16—C17—H17	119.7	O2—P1—C7	102.44 (16)
C19—C18—C17	118.8 (4)	O3—P1—C7	103.59 (17)
			
C6—C1—C2—C3	−0.5 (7)	C22—C21—C26—C25	0.1 (11)
Br1—C1—C2—C3	178.6 (4)	C26'—C21—C26—C25	60.1 (11)
C1—C2—C3—C4	0.1 (8)	C14—C21—C26—C25	179.0 (7)
C2—C3—C4—C5	0.9 (8)	C24—C25—C26—C21	0.8 (15)
C3—C4—C5—C6	−1.5 (7)	C26—C21—C22'—C23'	70 (2)
C2—C1—C6—N1	−178.6 (4)	C22—C21—C22'—C23'	−55 (2)
Br1—C1—C6—N1	2.3 (5)	C26'—C21—C22'—C23'	15 (3)
C2—C1—C6—C5	−0.2 (6)	C14—C21—C22'—C23'	−180 (2)
Br1—C1—C6—C5	−179.2 (3)	C21—C22'—C23'—C24	−1 (4)
C4—C5—C6—N1	179.6 (4)	C25—C24—C23'—C22'	−69 (2)
C4—C5—C6—C1	1.2 (6)	C25'—C24—C23'—C22'	−16 (3)
N1—C7—C12—C13	74.8 (5)	C23—C24—C23'—C22'	56 (3)
P1—C7—C12—C13	−50.7 (5)	C25—C24—C25'—C26'	63 (2)
N1—C7—C12—C14	−109.5 (4)	C23—C24—C25'—C26'	−63.4 (19)
P1—C7—C12—C14	125.0 (4)	C23'—C24—C25'—C26'	17 (3)
C14—C12—C13—N2	−0.3 (4)	C24—C25'—C26'—C21	−2 (3)
C7—C12—C13—N2	176.3 (3)	C22'—C21—C26'—C25'	−14 (2)
C13—C12—C14—N3	0.9 (4)	C26—C21—C26'—C25'	−60.1 (17)
C7—C12—C14—N3	−175.6 (3)	C22—C21—C26'—C25'	67.1 (17)
C13—C12—C14—C21	179.0 (4)	C14—C21—C26'—C25'	−178.8 (15)
C7—C12—C14—C21	2.5 (6)	C1—C6—N1—C7	−166.8 (3)
C20—C15—C16—C17	−2.9 (7)	C5—C6—N1—C7	14.8 (5)
N2—C15—C16—C17	176.3 (4)	C12—C7—N1—C6	136.8 (3)
C15—C16—C17—C18	−0.5 (8)	P1—C7—N1—C6	−99.9 (4)
C16—C17—C18—C19	3.0 (8)	C12—C13—N2—N3	−0.4 (4)
C17—C18—C19—C20	−2.1 (8)	C12—C13—N2—C15	−174.3 (3)
C16—C15—C20—C19	3.7 (7)	C16—C15—N2—C13	9.9 (6)
N2—C15—C20—C19	−175.5 (4)	C20—C15—N2—C13	−170.8 (4)
C18—C19—C20—C15	−1.2 (7)	C16—C15—N2—N3	−163.5 (4)
N3—C14—C21—C22'	−32.5 (14)	C20—C15—N2—N3	15.7 (5)
C12—C14—C21—C22'	149.6 (13)	C12—C14—N3—N2	−1.1 (4)
N3—C14—C21—C26	38.8 (7)	C21—C14—N3—N2	−179.4 (3)
C12—C14—C21—C26	−139.2 (5)	C13—N2—N3—C14	1.0 (4)
N3—C14—C21—C22	−142.3 (4)	C15—N2—N3—C14	175.4 (3)
C12—C14—C21—C22	39.7 (6)	C9—C8—O2—P1	−179.0 (5)
N3—C14—C21—C26'	131.9 (9)	C11—C10—O3—P1	−132.9 (5)
C12—C14—C21—C26'	−46.1 (10)	C8—O2—P1—O1	−49.5 (5)
C22'—C21—C22—C23	54.7 (10)	C8—O2—P1—O3	77.4 (4)
C26—C21—C22—C23	−0.4 (9)	C8—O2—P1—C7	−175.6 (4)
C26'—C21—C22—C23	−62.7 (9)	C10—O3—P1—O1	−24.8 (4)
C14—C21—C22—C23	−179.3 (5)	C10—O3—P1—O2	−151.6 (4)
C21—C22—C23—C24	−0.3 (10)	C10—O3—P1—C7	102.2 (4)
C25—C24—C23—C22	1.3 (13)	N1—C7—P1—O1	−66.3 (3)
C25'—C24—C23—C22	66.3 (11)	C12—C7—P1—O1	59.4 (3)
C23'—C24—C23—C22	−55.0 (13)	N1—C7—P1—O2	60.4 (3)
C25'—C24—C25—C26	−64.8 (13)	C12—C7—P1—O2	−173.9 (2)
C23—C24—C25—C26	−1.5 (16)	N1—C7—P1—O3	166.2 (2)
C23'—C24—C25—C26	70.1 (13)	C12—C7—P1—O3	−68.2 (3)
C22'—C21—C26—C25	−71.2 (12)		

### Hydrogen-bond geometry (Å, º)

**Table:**

*D*—H···*A*	*D*—H	H···*A*	*D*···*A*	*D*—H···*A*
N1—H1···O1^i^	0.86	2.48	3.305 (4)	162

Symmetry code: (i) −*x*+1, −*y*+1, −*z*.
